# The Aryl Hydrocarbon Receptor Is Expressed in Thyroid Carcinoma and Appears to Mediate Epithelial-Mesenchymal-Transition

**DOI:** 10.3390/cancers12010145

**Published:** 2020-01-07

**Authors:** Sonia Moretti, Nicole Nucci, Elisa Menicali, Silvia Morelli, Vittorio Bini, Renato Colella, Martina Mandarano, Angelo Sidoni, Efisio Puxeddu

**Affiliations:** 1 Department of Medicine, University of Perugia, Piazza Lucio Severi 1, 06132 Perugia (PG), Italy; sonia.moretti@unipg.it (S.M.); nicolenucci91@hotmail.it (N.N.); elisa.menicali@hotmail.it (E.M.); silviettamorelli@gmail.com (S.M.); vittorio.bini@unipg.it (V.B.); 2 Department of Experimental Medicine, University of Perugia, Piazza Lucio Severi 1, 06132 Perugia (PG), Italy; renato.colella75@gmail.com (R.C.); mandaranomartina@gmail.com (M.M.); angelo.sidoni@unipg.it (A.S.)

**Keywords:** AhR, thyroid cancer, EMT, SLUG, cell migration and invasiveness

## Abstract

Aryl hydrocarbon receptor (AhR) is expected to promote initiation, progression and invasion of cancer cells regulating proliferation, differentiation, gene expression, inflammation, cell motility and migration. Furthermore, an immunosuppressant function of AhR has been recognized. This study evaluated AhR expression and its role in thyroid cancer progression. AhR expression was assessed by qPCR in 107 thyroid cancer samples (90 PTCs, 11 MTCs, 6 ATCs), and by immunohistochemistry in 41 PTCs. To estimate receptor activation, the expression of target genes CYP1A1 and CYP1B1 was measured. AhR functional effects were evaluated in kynurenine-stimulated FTC-133 and BcPap cell lines by analyzing the expression of genes involved in EMT and cell motility. AhR mRNA expression resulted significantly higher in all the analyzed thyroid cancer samples compared to normal thyroid and a statistically significant correlation with CYP1B1 was detected. Kynurenine-stimulated FTC-133 and BcPap showed the activation of a specific AhR-driven EMT program characterized by E-cadherin decrease and SLUG, N-cadherin and fibronectin increase, resulting in boost of cell motility and invasion. This study confirmed the importance of the IDO1-Kyn-AhR pathway in thyroid cancer tumorigenesis, suggesting an AhR pivotal role in mediating an immunosuppressive microenvironment and favoring the acquisition of a mesenchymal phenotype that could promote invasiveness and metastasis.

## 1. Introduction

The Aryl hydrocarbon Receptor (AhR) is a cytosolic ligand-activated transcription factor, originally characterized as an important player of detoxification pathways for xenobiotics and environmental pollutants such as polycyclic aromatic hydrocarbons (PAHs) and halogenated aromatic hydrocarbons (HAHs). To date, more than 400 exogenous ligands have been identified including 2, 3, 7, 8-tetrachlorodibenzo-p-dioxin (TCDD), the most powerful AhR agonist [1]. As a result of ligand binding, AhR dissociates from a chaperone complex (Heat Shock Protein 90, Prostaglandin E Synthase 3 (p23) and hepatitis B virus X-associated protein (XAP2), also known as aryl hydrocarbon receptor interacting protein (AIP), heterodimerizes with the Aryl Hydrocarbon Nuclear Translocator (ARNT), and translocates into the nucleus. The AhR/ARNT complex binds xenobiotic or dioxin-responsive elements (XRE or DRE) regions, which are enhancer DNA elements located in the 5′-flanking region of target genes, inducing their transcription. Among those, cytochrome P450 1A1 (CYP1A1) and cytochrome P450 1B1 (CYP1B1) are almost totally dependent on AhR activity and are implicated in detoxification of xenobiotics. AhR is highly conserved across different species [2] and constitutively expressed practically in all tissue, especially placenta, liver and lungs.

The discovery that the receptor is involved in immune responses [3,4] and carcinogenesis [5,6] led to an increasing interest towards AhR. In fact, recent studies have shown that AhR is overexpressed and/or constitutively active, even in the absence of environmental ligands, in several human tumors such as breast, liver, lung, gastric, pancreatic, prostate, urothelial, ovarian cancers, T-cell leukemia, glioma and medulloblastoma [6,7] and that it can act as a promoter [8] or a suppressor of cancers growth [9,10]. Moreover, epidemiological and experimental animal data provide evidence for an association between AhR and cancer initiation and progression. In fact, exposure to PAHs led to the development of a variety of cancers, whose initiation appeared to be mediated by the receptor, in humans or mice [5,6].

Furthermore, AhR is able to activate the human embryonic protein SNAI2, commonly known as SLUG [11], a master repressor of E-cadherin transcription [12], suggesting a pivotal role of the receptor in the induction and/or regulation of the epithelial to mesenchymal transition (EMT) [13].

In gliomas, kynurenine, the product of the enzymatic reaction catalyzed by Indoleamine 2,3-Dioxygenase 1 (IDO1), Indoleamine 2,3-Dioxygenase 2 (IDO2) and Tryptophan 2,3-Dioxygenase (TDO), was shown to be an endogenous ligand of human AhR and to suppress antitumor immune responses and to promote tumor cell survival and motility through AhR in an autocrine/paracrine fashion [8].

We previously demonstrated that IDO1 has an important role in thyroid carcinogenesis inducing an immunosuppressant tumor microenvironment [14]. Moreover, other studies demonstrated that papillary thyroid carcinomas overexpressed AhR with higher intensity in BRAF mutated samples [15,16].

To deeper investigate the role of the IDO1-Kynurenine-AhR pathway in thyroid cancer pathogenesis, we analyzed AhR expression in human-derived thyroid cancers and in thyroid tumors from BRAF-transgenic mice. Furthermore, we evaluated the AhR-mediated transcriptional and functional effects of kynurenine in two human thyroid cancer cell lines. The obtained results suggest that AhR is implicated in thyroid cancer initiation and progression both promoting the establishment of an immunosuppressive tumor microenvironment and EMT.

## 2. Results

### 2.1. AhR Is Expressed and Functionally Activated in Thyroid Cancer Samples

**Figure 1:**
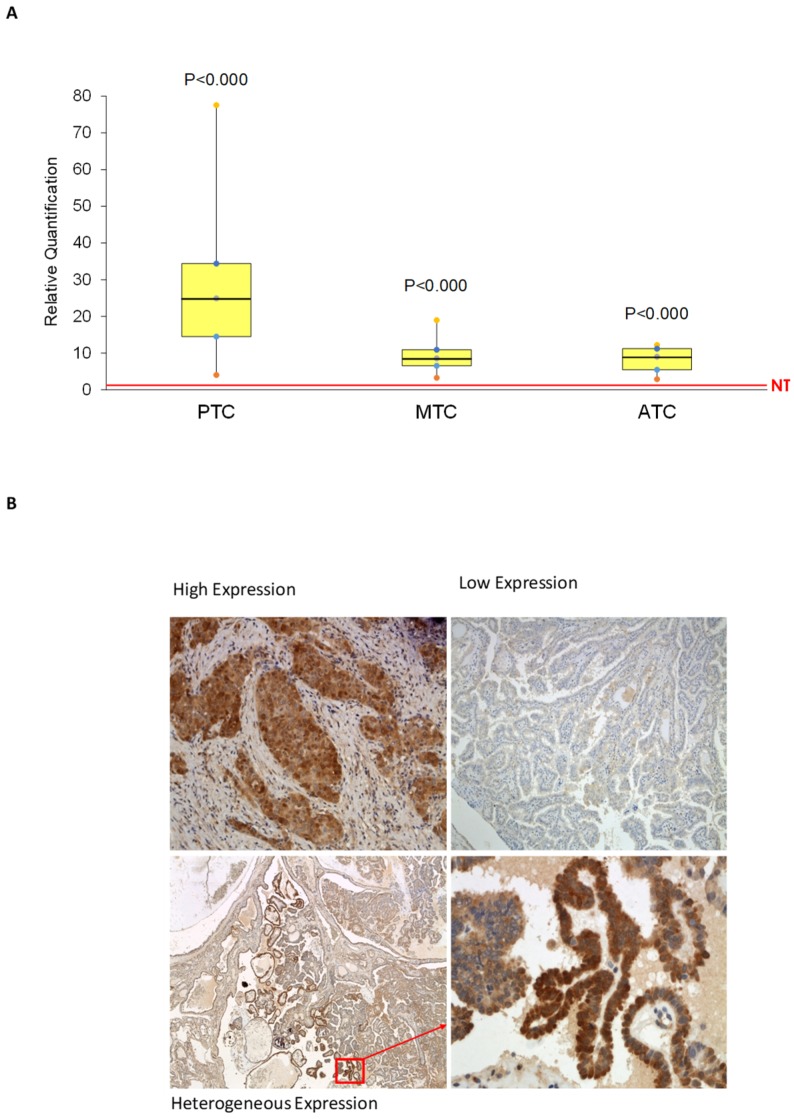
AhR expression in thyroid cancer samples. (**A**) After total RNA extraction from thyroid cancer samples and cDNA synthesis, AhR mRNA expression was evaluated by qPCR. The data are presented as medians of Relative Quantification (RQ) obtained normalizing the acquired data with those of the normal thyroid sample, and *p* values were calculated using the one-sample *t* test. In all 107 analyzed samples, AhR expression results higher compared with normal thyroid (median difference PTCs: 24.90 (range 4.034–77.56, *p* ≤ 0.0001), MTCs: 8.55 (range 3.29–18.97, *p* ≤ 0.0001), ATCs: 9.02 (range 2.89–12.20, *p* ≤ 0.0001). The yellow boxes depict the values in the second and third quartiles. The black segment inside the boxes indicates the median. The vertical bars outside the boxes indicate the ranges. The horizontal red line refers to normal thyroid (NT). (**B**) AhR expression was evaluated by IHC on tissue sections of 41 PTC cases with a primary monoclonal anti-human AhR antibody. AhR immunostaining showed an increased expression of the receptor in the cancerous epithelial cells of the PTCs with 3 different staining patterns: high expression (left top panel, 200×), low expression (right top panel, 100×), and heterogeneous expression (bottom panels, 40× and 400×).

AhR expression was evaluated by quantitative polymerase chain reaction (qPCR) in 90 papillary thyroid cancers (PTCs), 11 medullary thyroid cancers (MTCs), and 6 anaplastic thyroid cancers (ATCs) and by immunohistochemistry (IHC) in a subgroup of 41 of the 90 PTCs. A commercially available cDNA derived from a mix of 65 normal thyroid samples (BD Biosciences CLONTECH) was used as reference. AhR mRNA expression was higher than normal thyroid in all the analyzed samples. In detail, in PTC the median difference was 24.90 (range 4.034–77.56, *p* ≤ 0.0001), in MTC 8.55 (range 3.29–18.97, *p* ≤ 0.0001), in ATC 9.02 (range 2.89–12.20, *p* ≤ 0.0001) (Figure 1A).

AhR immunostaining showed expression of the receptor in the cancerous epithelial cells of the PTCs. In most cases, it resulted higher than in adjacent normal thyroid tissue. Interestingly, AhR IHC showed three different patterns: high expression, low expression and heterogeneous expression. An enhancement of the staining in the infiltrative areas was observed in about half of the analyzed samples (Figure 1B).

No significant correlation could be found between AhR mRNA expression levels and IHC score.

The level of AhR functional activation was evaluated by measuring CYP1A1 and CYP1B1 mRNA expression in the thyroid cancer samples. CYP1A1 was undetectable in normal and tumor samples, whereas CYP1B1 expression was significantly higher in PTC than in normal thyroid with median difference of 1.27 (range 0.10–20.87, *p* = 0.0004). Conversely, in MTC and in ATC CYP1B1 expression was lower than normal thyroid (MTC: median difference 0.068 [range 0.009–0.44, *p* < 0.0001]; ATC: median difference 0.06 [range 0.016–1.91, *p* = 0.0034]). Correlation between AhR and CYP1B1 mRNA expression levels showed a positive statistically significant association (Spearman’s rho 0.431, *p* <0.0001).

PTCs harboring BRAFV600E mutation (65/90; 72.2%) showed significantly higher AhR mRNA expression levels compared to BRAF wild type (WT) (25/90; 27.8%) PTCs (BRAFV600E: median: 27.0, min: 4.56, max: 77.55; BRAF WT: median: 16.91, min: 4.03, max: 41.38; *p* = 0.03). AhR expression levels in BRAF WT PTC samples were higher than in MTC and ATC (BRAF WT: median: 16.91, min: 4.03, max: 41.38; MTC: median: 8.55 min: 3.29, max: 18.97; ATC: median: 9.02, min: 2.89, max: 12.20).

### 2.2. AhR Is Overexpressed in BRAFV600E-Harboring Murine Thyroid Cancer Tissue

**Figure 2:**
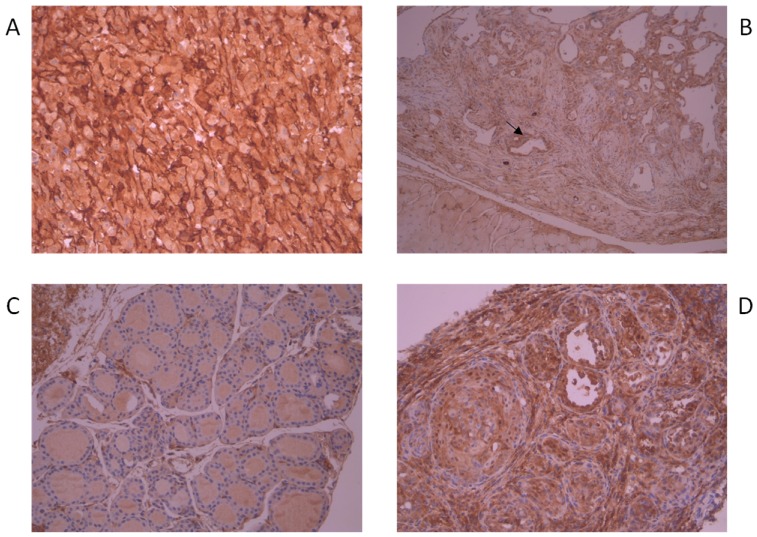
AhR expression in thyroid samples of transgenic mice. AhR expression was evaluated by IHC on tissue sections of 20 tissues (14 thyroid cancers, 4 normal thyroids and 2 lymph node metastases) with a primary monoclonal anti- mouse AhR antibody. (**A**) High expression of AhR in a thyroid cancer from a TetOn-BRAF-P53 mouse (200×). (**B**) Heterogeneous AhR expression in a thyroid tumor from a BRAF-Lox/TPO-Cre mouse (100×). Arrow: infiltrative cells with higher AhR staining. (**C**) AhR negative staining in a thyroid derived from a Tg-rtTA/tetO-BRAFV600E mouse not treated with dox to induce BRAFV600E expression (200×). (**D**) AhR positive staining in a thyroid derived from a Tg-rtTA/tetO-BRAFV600E mouse treated for 7 days with dox to induce BRAFV600E expression (200×).

We evaluated AhR expression in thyroid cancer samples derived from transgenic mice characterized by conditional expression of BRAFV600E in the thyroid. AhR was measured by IHC in 14 thyroid cancers, 4 normal thyroids and 2 lymph node metastases derived from 3 different mouse models kindly provided by Dr. Jeffrey Knauf (Memorial Sloan Kettering Cancer Center, New York) [17]. In detail, we analyzed 6 thyroid tumors and 2 lymph node metastases from mice characterized by thyroid doxycycline (dox)-inducible BRAFV600E expression in a p53-/- background (TetOn-BRAF-P53), treated for 6–10 weeks with dox to induce BRAFV600E expression. All analyzed tumor samples showed higher AhR staining compared to adjacent normal thyroid and AhR was clearly detectable in the 2 lymph node metastasis, too. Figure 2A shows an example of AhR staining in one of these tumors with high and uniform AhR expression (Figure 2A). Similarly, AhR staining of 6 thyroids derived from BRAFV600E knock-in mice (BRAF-Lox/TPO-Cre) showed higher staining in tumors compared with normal tissues. Some of these tumors had heterogeneous AhR expression with an enrichment in infiltrative areas (Figure 2B, arrow). The 3 normal thyroids, derived from mice BRAF-Lox/TPO-Cre homozygous for BRAF wild type (WT), were negative for AhR. Finally, to study the first stages of BRAFV600E-mediated transformation, we analyzed 2 thyroids from Tg-rtTA/tetO-BRAFV600E mice treated for 7 days with dox and 1 control thyroid from an untreated animal. These mice are characterized by dox-inducible BRAFV600E expression in the thyroid. The control thyroid did not expressed AhR (Figure 2C), whereas AhR staining was evident in the 2 samples in which BRAFV600E expression was induced by 7 days of dox-treatment (Figure 2D).

### 2.3. AhR Is Expressed and Functionally Activated in FTC-133 and Bcpap Cell Lines

**Figure 3:**
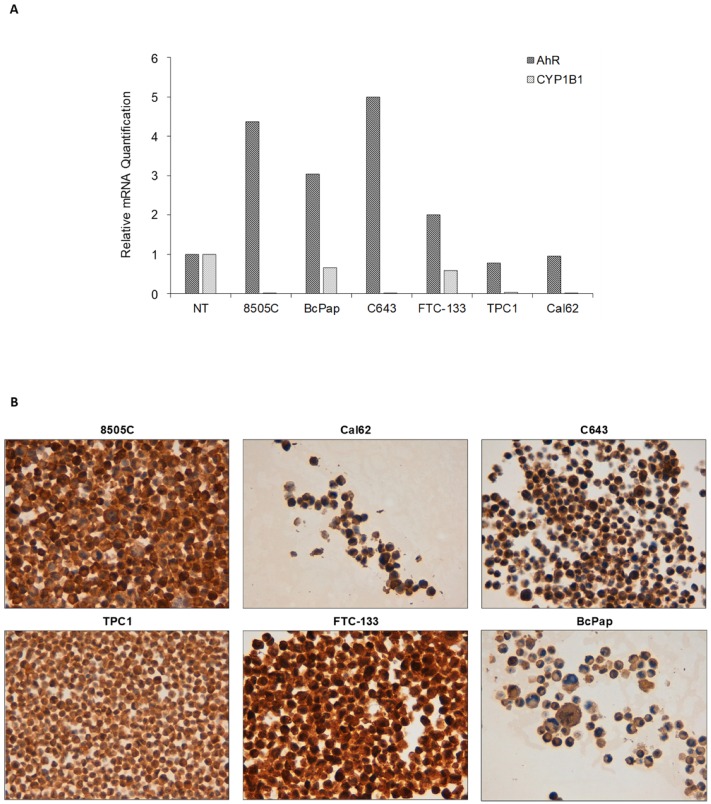
AhR expression in thyroid cancer cell lines. (**A**) After total RNA extraction from thyroid cancer cell lines and cDNA synthesis, AhR and CYP1B1 mRNA expressions were evaluated by qPCR. The data are presented as means of RQ obtained normalizing the acquired data with those of the normal thyroid sample. Only FTC-133 and BcPap cell lines showed detectable CYP1B1 mRNA, although lower than normal thyroid. (**B**) AhR expression was evaluated by ICC on cell pellets using a primary monoclonal anti-human AhR antibody. Immunostaining showed that AhR was localized prevalently in the cytoplasm in all the cell lines except for FTC-133 where a simultaneous cytoplasmic and nuclear staining was detected. Magnification: 400×.

After having evaluated expression and activation state of AhR in human thyroid cancer samples, we analyzed AhR and CYP1B1 expression in human thyroid carcinoma cell lines. In detail, AhR mRNA expression levels resulted higher than normal thyroid in C643 (5.0 fold), 8505C (4.4 fold), BcPap (3.0 fold) and FTC-133 (2.0 fold) cell lines and lower than in normal thyroid in TPC1 (0.8 fold) and Cal62 (0.9 fold) cell lines (Figure 3A). CYP1B1 mRNA expression levels resulted undetectable in C643, very low in 8505C, Cal62, TPC1 (0.005, 0.005 and 0.03 fold respectively), and higher, although still lower than normal thyroid, in FTC-133 (0.6 fold) and BcPap (0.7 fold) cell lines (Figure 3A). AhR immunocytochemistry (ICC) analysis of cell line pellets showed high staining in 8505C and FTC-133 cell lines (ICC score = 3), intermediate staining in Cal62, C643 and TPC1 (ICC score = 2) and low/intermediate staining in BcPap (ICC score = 1 in cytoplasm and 2 in membrane). Interestingly, only in FTC-133 cells AhR was also located within the nucleus, suggesting its activation even in the absence of exogenous ligand (Figure 3B).

### 2.4. Kynurenine Activates AhR in Thyroid Cancer Cell Lines

To evaluate the functional effects of AhR activation in thyroid carcinomas, we selected the FTC-133 and BcPap cell lines for further studies. Indeed, both these cell lines showed basal IDO1 [14] and CYP1B1 expression. Moreover, in the case of FTC-133, an increase in kynurenine secretion in the conditioned medium could also be detected [14]. These data indicated a potential responsiveness to kynurenine stimulation.

**Figure 4:**
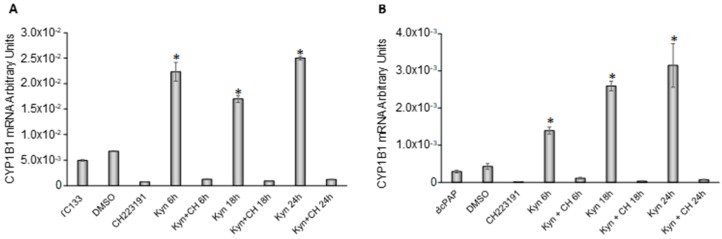
Kynurenine induces CYP1B1 expression in thyroid cancer cell lines. FTC-133 (**A**) and BcPap cells (**B**) were incubated for the indicated times with kynurenine (100 µM) in the presence or absence of the AhR inhibitor CH223191 (10 µM) and harvested for total RNA extraction and cDNA synthesis. CYP1B1 mRNA expression levels were assayed by qPCR. The data are presented as means of arbitrary units. All experiments were repeated three times and the more representative results are depicted. DMSO: Dimethyl sulfoxide treated cells; Kyn: kynurenine treated cells; CH: CH223191 treated cells. *p* values were calculated applying the unpaired Student’s *t* test. *, *p* < 0.05.

In the first instance, we evaluated the ability of kynurenine to activate AhR by analyzing kynurenine-induced CYP1B1 expression. In FTC-133 cells, the administration of kynurenine stimulated a significant increase of CYP1B1 that started after 6 h of treatment and lasted throughout the experiment (4.5 fold at 6 h, *p* = 0.005; 3.4 fold at 18 h, *p* = 0.001; 5.1 fold at 24 h, *p* = 0.0002) (Figure 4A).

Simultaneous administration of CH223191 [18], an inhibitor of AhR nuclear translocation, impaired the kynurenine-induced increase of the cytochrome expression (Figure 4A). Similarly, in BcPap kynurenine caused an increase in CYP1B1 mRNA level (4.8 fold at 6 h, *p* = 0.003; 8.9 fold at 18 h, *p* = 0.001; 10.9 fold at 24 h, *p* = 0.01) which was impaired by the co-administration of CH223191 (Figure 4B).

### 2.5. Kynurenine Induces IDO1 and SLUG Expression through AhR

**Figure 5:**
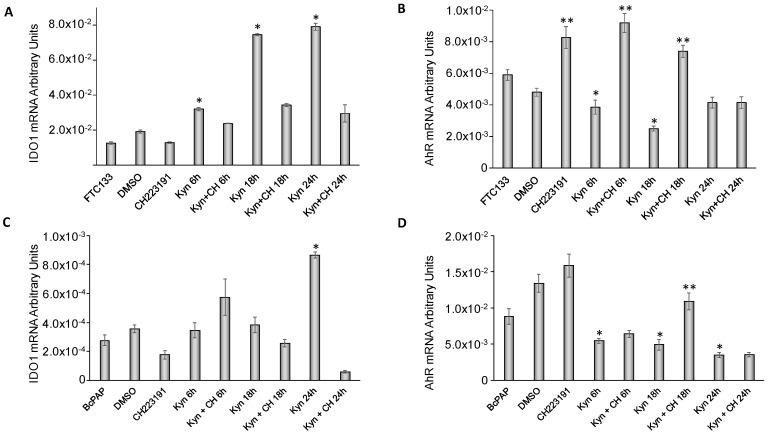
Effects of kynurenine on immune regulatory genes IDO1 and AhR. FTC-133 and BcPap cells were incubated for the indicated times with kynurenine (100 µM) in the presence or absence of the AhR inhibitor CH223191 (10 µM) and harvested for total RNA extraction and cDNA synthesis. IDO1 and AhR mRNA expression levels were assayed by qPCR. The data are presented as means of arbitrary units. All experiments were repeated three times and the more representative results are depicted. *p* values were calculated applying the unpaired Student’s *t* test. DMSO: Dimethyl sulfoxide treated cells; Kyn: kynurenine treated cells; CH: CH223191 treated cells. (**A**) IDO1 expression in FTC-133 cells; (**B**) AhR expression in FTC-133 cells; (**C**) IDO1 expression in BcPap cells; (**D**) AhR expression in BcPap cells. * Differences between kynurenine-treated cells and untreated cells, *p* < 0.05. ** Differences between cells treated with CH223191 alone or in combination with kynurenine and respective controls, *p* < 0.05.

After demonstrating that kynurenine activates AhR, we investigated the effect of AhR activation on genes involved in immune tolerance (IDO1 and AhR itself) and in EMT (SLUG). As shown in Figure 5A, in FTC-133 cells kynurenine treatment induced an increase in IDO1 mRNA levels (1.7 fold at 6 h, *p* = 0.007; 3.9 fold at 18 h, *p* = 0.0002 and 4.1 fold at 24 h, *p* < 0.0001) compared to the control. Treatment with the AhR inhibitor CH223191 abolished this effect (Figure 5A). Conversely, treatment with kynurenine induced a significant decrease in AhR expression levels (0.8 fold at 6 h, *p* = 0.04; 0.5 fold at 18 h, *p* = 0.004), while treatment with CH223191 either alone or in combination with kynurenine induced a rise in the receptor mRNA levels (CH223191 alone vs. DMSO: 1.7 fold, *p* = 0.04; CH223191 + kynurenine 6 h vs. kynurenine 6 h: 2.4 fold, *p* = 0.001; CH223191 + kynurenine 18 h vs. kynurenine 18 h: 2.9 fold, *p* = 0.003). The effects of the treatments were lost at 24 h (Figure 5B).

Similarly, in BcPap cells, kynurenine alone induced a significant IDO1 mRNA increase at 24 h (3.13 folds, *p* = 0.001), whereas the co-administration of kynurenine and CH223191 reduced kynurenine-induced IDO1 expression (Figure 5C). In BcPap cells, treatment with kynurenine alone reduced significantly AhR expression levels (0.62 fold at 6 h, *p* = 0.04; 0.56 fold at 18 h, *p* = 0.013 and 0.4 fold at 24 h, *p* = 0.015) while they increased significantly in presence of CH223191, after 18 h (2.2 fold, *p* = 0.02) (Figure 5D).

**Figure 6:**
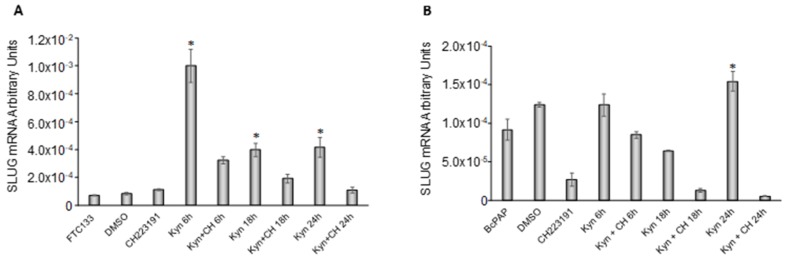
Kynurenine induces SLUG expression. FTC-133 (**A**) and BcPap (**B**) cells were incubated for the indicated times with kynurenine (100 µM) in the presence or absence of the AhR inhibitor CH223191 (10 µM) and harvested for total RNA extraction and cDNA synthesis. SLUG mRNA expression levels were assayed by qPCR. The data are presented as means of arbitrary units. All experiments were repeated three times and the more representative results are depicted. DMSO: Dimethyl sulfoxide treated cells; Kyn: kynurenine treated cells; CH: CH223191 treated cells. *p* values were calculated applying the unpaired Student’s *t* test. *, *p* < 0.05.

In the FTC-133 cell line, kynurenine induced a significant increase of SLUG mRNA that started at 6 h of treatment and lasted for all the duration of the experiment (12 fold at 6 h, *p* = 0.006; 4.8 fold at 18 h, *p* = 0.008 and 5.0 fold at 24 h, *p* = 0.01). CH223191 reverted, partially or totally, this effect (Figure 6A). In BcPap cells, the increase of SLUG mRNA levels induced by kynurenine was only significant at 24 h (1.7 fold, *p* = 0.05). Combined administration of CH223191 and kynurenine abolished this effect (Figure 6B).

**Figure 7:**
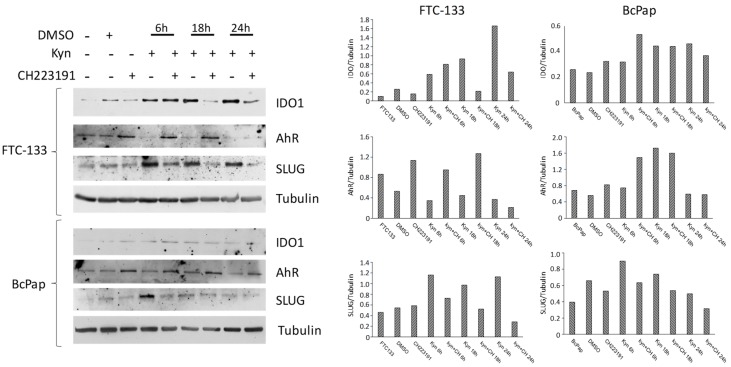
Effects of kynurenine on IDO1, AhR and SLUG protein levels. FTC-133 and BcPap cells were incubated for the indicated times with kynurenine (100 µM) in the presence or absence of the AhR inhibitor CH223191 (10 µM) and harvested for protein extraction. Expression of IDO1, AhR, SLUG and tubulin were assayed by immunoblot. All the experiments were repeated twice, and the more representative results are depicted. The bands of the shown experiment were analyzed by densitometry and normalized with tubulin. DMSO: Dimethyl sulfoxide treated cells; Kyn: kynurenine treated cells; CH: CH223191 treated cells.

To confirm at the protein level the effects induced by kynurenine on gene expression, we performed a western blotting analysis in FTC-133 and BcPap cell lines (Figure 7). In FTC-133 cells, treatment with kynurenine induced an increase of IDO1 and SLUG expressions that started at 6 h and lasted for the entire duration of the experiment. On the contrary, co-administration of CH223191 abolished the kynurenine effect. Conversely, AhR was barely detectable in cells treated with kynurenine but administration of CH223191 led to its increase. In BcPap cells, treatment with kynurenine induced a slight increase of SLUG expressions at 6 h but no effect on IDO1 expression. Co-administration of CH223191 abolished the kynurenine effect on SLUG. Conversely, the AhR expression was not stimulated by kynurenine treatment but increased by the addiction of CH223191.

### 2.6. Kynurenine Mediates EMT

**Figure 8:**
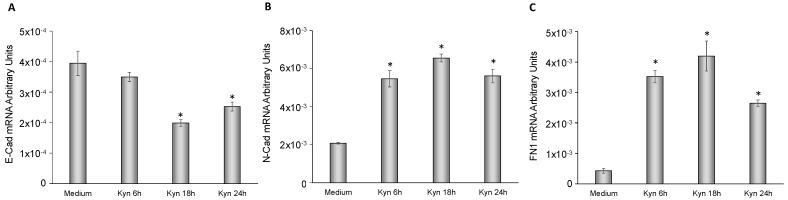
Effect of kynurenine on EMT markers in FTC-133. FTC-133 cells were incubated for the indicated times with kynurenine (100 µM) and harvested for total RNA extraction and cDNA synthesis. E-cadherin (**A**), N-cadherin (**B**), and fibronectin-1 (**C**) mRNA expression levels were assayed by qPCR. The data are presented as means of arbitrary units. All experiments were repeated three times and the more representative results are depicted. Kyn: kynurenine treated cells. *p* values were calculated applying the unpaired Student’s *t* test. *, *p* < 0.05.

To more deeply investigate the role of kynurenine in EMT, we analyzed the mRNA expression levels of E-cadherin, N-cadherin and fibronectin-1 in FTC-133 and BcPap cell lines. When FTC-133 cells were treated with kynurenine, E-cadherin mRNA halved after 18 h (*p* = 0.0168 Figure 8A) while N-cadherin and fibronectin-1 significantly increased at 6 h of treatment and lasted for the whole duration of the experiment (N-cadherin: 2.62 fold at 6 h, *p* = 0.005; 3.14 fold at 18 h, *p* = 0.0008; 2.7 fold at 24 h, *p* = 0.003. Fibronectin-1: 8.25 fold at 6 h, *p* = 0.002; 9.8 fold at 18 h, *p* = 0.006; 6.2 fold at 24 h, *p* = 0.0001) (Figure 8B,C).

**Figure 9:**
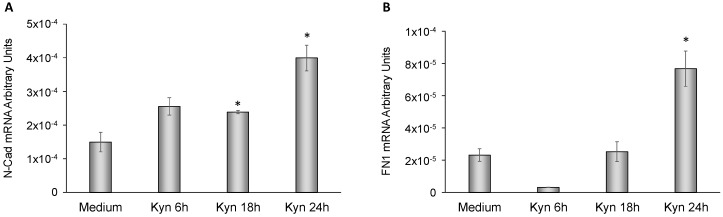
Effect of kynurenine on EMT markers in BcPap. BcPap cells were incubated for the indicated times with kynurenine (100 µM) and harvested for total RNA extraction and cDNA synthesis. N-cadherin (**A**) and fibronectin-1 (**B**) mRNA expression levels were assayed by qPCR. The data are presented as means of arbitrary units. All experiments were repeated three times and the more representative results are depicted. Kyn: kynurenine treated cells. *p* values were calculated applying the unpaired Student’s *t* test. *, *p* < 0.05.

In BcPap cell line, E-cadherin was undetectable in basal condition and after adding kynurenine, whereas N-cadherin and fibronectin-1 were significantly increased at 18 h and/or 24 h (N-cadherin: 1.6 fold at 18 h, *p* = 0.03; 2.7 fold at 24 h, *p* = 0.009. Fibronectin-1: 3.3 fold at 24 h, *p* = 0.01) (Figure 9A,B).

**Figure 10:**
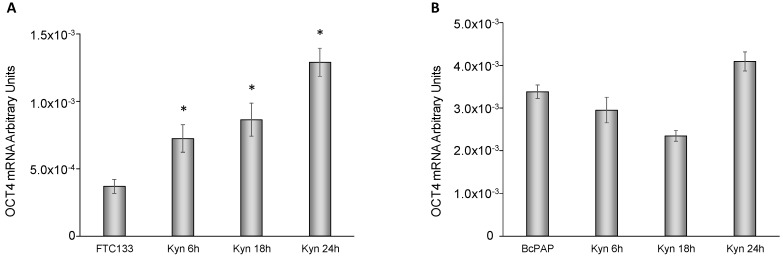
Kynurenine induced OCT4 gene. FTC-133 (**A**) and BcPap (**B**) cells were incubated for the indicated times with kynurenine (100 µM) and harvested for total RNA extraction and cDNA synthesis. OCT4 mRNA expression levels were assayed by qPCR. The data are presented as means of arbitrary units. All experiments were repeated three times, and the more representative results are depicted. Kyn: kynurenine treated cells. *p* values were calculated applying the unpaired Student’s *t* test. *, *p* < 0.05.

Finally, we investigated the effect of kynurenine administration on expression levels of octamer-binding transcription factor 4 (OCT4), one of the master genes of stemness. In FTC-133 cells kynurenine induced a significant increase of OCT4 mRNA expression for all the duration of the experiment (2.0 fold at 6 h, *p* = 0.01; 2.3 fold at 18 h, *p* = 0.03; 3.5 fold at 24 h, *p* = 0.008), whereas in BcPap cells kynurenine did not modified significantly OCT4 expression levels (Figure 10A,B).

### 2.7. Cell Migration Assay

**Figure 11:**
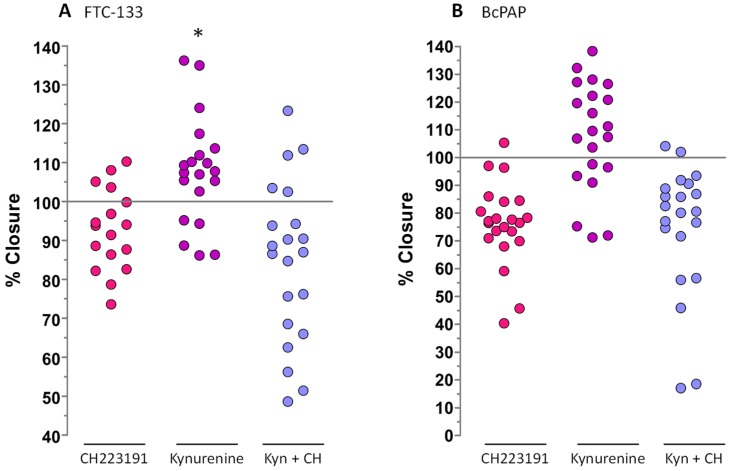
Wound healing assay. FTC-133 and BcPap cells were seeded in 96 well plates and, at confluence, stoppers were removed, and cells treated with kynurenine (100 µM) and/or CH223191 (10 µM). Images were acquired and the free cells area was measured with ImageJ software. The data are presented as means of percentage of wound closure obtained normalizing the free cells area of each sample with the free cells area of untreated cells. Data of three independent experiments, in which each samples was repeated six times, were collected. Kyn: kynurenine treated cells; CH: CH223191 treated cells. (**A**) FTC-133 cell; (**B**) BcPap cell. *p* values were calculated applying the one sample Student’s *t* test. *, *p* < 0.05.

To evaluate kynurenine-induced effect on cell migration, we performed a wound assay. Kynurenine induced a significant increase of FTC-133 cells capability to close the wound compared to untreated cells (*p* = 0.024). BcPap cells treated with kynurenine exhibited an increased ability to close the wound too, but in this cell line the difference with parental cells was not significant (*p* = 0.08). CH223191 abolished this effect in both cell lines (Figure 11).

**Figure 12:**
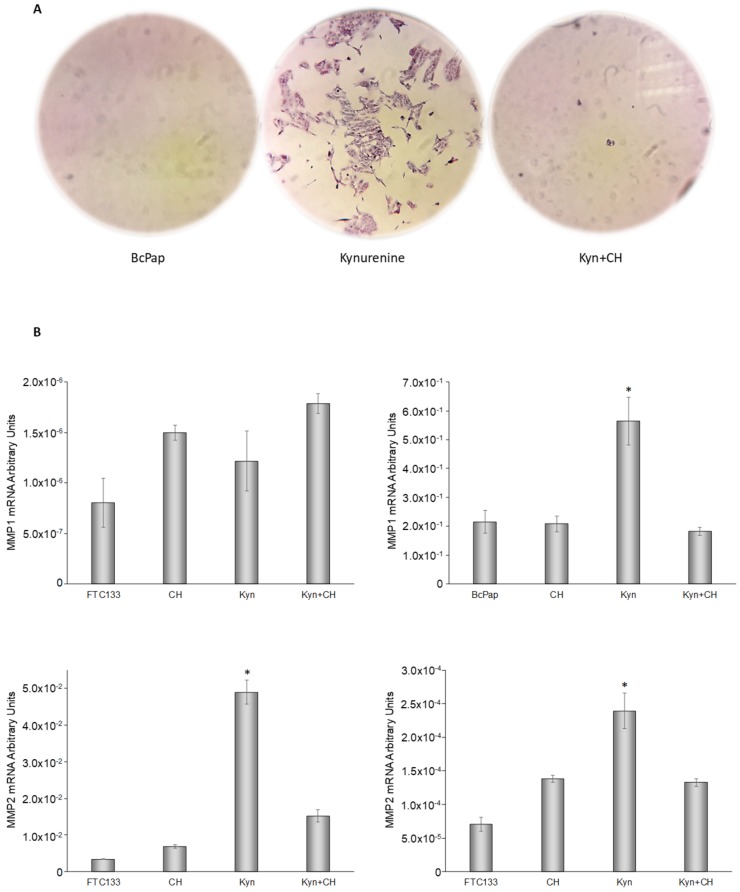
Kynurenine-induced AhR activation in tumor invasiveness. (**A**) Boyden chamber assay. FTC-133 and BcPap cells were seeded in a transwell in serum free medium. The bottom wells were filled with 10% FBS medium. Treatments were added to both chambers and renewed every 8 h. After 48 h, cells were fixed, stained with crystal violet and images were acquired using an optical microscope. Three independent experiments were performed and a representative one was shown. (**B**) FTC-133 and BcPAP cells were incubated for the indicated times with kynurenine (100 µM) in the presence or absence of the AhR inhibitor CH223191 (10 µM) and harvested for total RNA extraction and cDNA synthesis. MMP1 and MMP2 mRNA expression levels were assayed by qPCR. The data are presented as means of arbitrary units. All experiments were repeated three times and the more representative results are depicted. Kyn: kynurenine treated cells; CH: CH223191 treated cells. *p* values were calculated applying the unpaired Student’s *t* test. *, *p* < 0.05.

To evaluate the effect of kynurenine-induced AhR activation on cell invasiveness, we performed a Boyden chamber assay. Both cell lines were able to digest the extra-cellular matrix (ECM) and to reach the semipermeable membrane of the transwell, independently from the treatment. Interestingly, BcPap cells acquired the ability to migrate at the bottom of the 24 wells plate, after the administration of kynurenine. This feature was abolished by the simultaneous addition of CH223191 (Figure 12A).

Moreover, we investigated the expression of metalloproteases MMP1, MMP2 and MMP9 in the 2 cell lines. The results showed no expression of MMP9, both in basal condition and after kynurenine administration. MMP1 expression was 2 × 10^5^-fold higher in BcPap than in FTC-133. On the contrary, MMP2 was more expressed in FTC-133 than in BcPap (about 50 fold). In BcPap cell line MMP1 and MMP2 were up-regulated by kynurenine and CH223191 prevented their increase; while in FTC-133, only MMP2 showed this behavior (Figure 12B).

## 3. Discussion

We have previously demonstrated that IDO1 is up-regulated in thyroid cancers and contributes to the creation of an immunosuppressant environment [14]. The enzymatic cleavage of tryptophan induced by IDO1 produces kynurenine that was identified as an endogenous ligand of AhR [8]. To more deeply investigate the role of IDO1-kynurenine-AhR in thyroid cancer, we analyzed AhR expression in a collection of tissues (90 PTCs, 11 MTCs and 6 ATCs) as mRNA or, in a subgroup of 41 PTCs, as protein. Surprisingly, all analyzed tumor samples showed higher expression of AhR than normal thyroid at the mRNA level. Interestingly, PTC showed higher levels than MTC or ATC. This finding might be related to the fact that expression of AhR is correlated with the levels of differentiation of the tumors, dropping where differentiation gets lower. However, a sample size bias (90 PTC vs. 6 ATC or 11 MTC) may also account for it. Furthermore, those data were confirmed at the protein level too. Thus, the finding indicated an important role of the receptor in thyroid carcinogenesis. Unfortunately, we could not detect a statistically significant correlation between mRNA levels and IHC scores. This discrepancy was probably due to the heterogeneous expression of AhR observed in the majority of analyzed samples or to the low number of cases submitted to IHC. It is possible that by increasing the number of cases, a correlation could be found. Interestingly, AhR staining was more intense in cancer infiltrating cells, suggesting a role of the receptor in the invasion processes. As previously reported by other authors [15,16], we found a significant association between BRAF mutation and higher levels of AhR expression. No other significant association with the considered clinico-pathological features could be found.

To investigate if AhR overexpression in thyroid cancers was associated with the activation of its transcriptional activity, we evaluated the expression levels of AhR target gene CYP1B1. The results confirmed that in thyroid neoplasms the overexpression of the receptor was associated with a significant activation of its transcriptional activity. It is possible to speculate that in these cancers the expression of IDO1 and the synthesis of kynurenine might contribute to activate the receptor. As a confirmation, IHC staining for AhR showed stained nuclei, index of AhR nuclear migration and transcriptional activation, especially in the regions of tumor invasion.

We analyzed AhR expression in thyroid tissues of three different mice models characterized by conditional expression of BRAFV600E in the thyroid. These models recapitulated different stages of thyroid tumorigenesis. One model, characterized by a thyroid dox-inducible expression of BRAFV600E in a P53-/- genetic background (TetOn-BRAF-P53), developed high penetrant and poorly differentiated thyroid tumors that were similar to human poorly differentiated thyroid cancer (PDTC) and ATC. Conversely, BRAFV600E knock-in mice (BRAF-Lox/TPO-Cre) generated PTC-like cancers. Finally, the model characterized by a thyroid dox-inducible expression of BRAFV600E in a WT genetic background (Tg-rtTA/tetO-BRAFV600E), was useful to investigate the first stages of thyroid transformation induced by BRAFV600E. Interestingly, AhR expression in the tumors of the mice showed the same characteristic of human cancers. Indeed, all the tumors, regardless of the histotype, presented higher AhR expression compared to normal thyroid and AhR expression was generally heterogeneous with an enhancement in infiltrative cells. The observation that seven days of BRAFV600E induction were sufficient to obtain an increased expression of AhR, confirmed the importance of BRAF mutation signaling in AhR regulation.

Evaluation of AhR mRNA expression in human thyroid carcinoma cell lines showed overexpression of the receptor in four to six. However, CYP1B1 mRNA was detectable only in FTC-133 and BcPap cells suggesting that only in these lines AhR was transcriptional active in basal condition. Immunocytochemistry confirmed the presence of the receptor in the cytoplasm of all the positive cell lines. Conversely, only FTC-133 cells revealed a clear AhR nuclear staining. Interestingly, FTC-133 cells expressed high levels of IDO1, and the presence of kynurenine could be detected in the conditioned medium even in basal conditions [14]. Thus, it is possible to speculate that endogenously produced kynurenine might drive AhR activation in the absence of exogenous ligands.

The expression levels of CYP1A1 were also explored. Interestingly, they resulted undetectable both in thyroid cancer samples and in thyroid cancer-derived cell lines. This finding was consistent with the observation that CYP1A1 is prevalently involved in detoxification of carcinogens with a cancer preventive action [19], whereas CYP1B1 expression is elevated in a wide range of human tumors [20].

The significant increase of CYP1B1 expression induced by kynurenine treatment in FTC-133 and BcPap cell lines, confirmed the capability of kynurenine to activate AhR in these cell lines. Moreover, administration of CH223191, an inhibitor of AhR nuclear translocation, was efficiently able to prevent this increase, blocking the transcriptional effects induced by kynurenine on the receptor. After having validated the system, to better understand the functions of AhR in thyroid carcinogenesis, we evaluated the transcriptional effects of kynurenine-mediated activation of the receptor on genes involved in immune-tolerance and EMT. To confirm the specificity of AhR in the regulation of these genes, CH223191 was administrated in combination with kynurenine. We performed these experiments in FTC-133, characterized by high level of potentially active AhR, as demonstrated by the nuclear staining. Furthermore, the data were confirmed in the BcPap cell line, characterized by IDO1 overexpression, although kynurenine resulted undetectable in the basal conditioned medium [14].

As expected, kynurenine induced an increase in IDO1 mRNA levels that was completely reversed by the co-administration of the AhR inhibitor. Conversely, kynurenine did not appear to strongly influence AhR expression whereas the blockage of nuclear translocation induced by the treatment with CH223191 appeared to increase the transcription levels of AhR, probably due to a negative feed-back of the activated receptor on its transcription that is lost after the addition of the inhibitor. Interestingly, this effect occurred in both cell lines also in the absence of exogenous ligand. However, it was more pronounced in FTC-133 cells, probably because this cell line is characterized by a more active IDO1-kynurenine-AhR axis.

The presence of AhR in the infiltrating cancer cells and the knowledge that SLUG is one of the target gene of AhR [11], prompted us to investigate the effect of kynurenine in EMT. The obtained data confirmed that kynurenine induces SLUG expression through AhR. Indeed, the overexpression of SLUG was detected at both the mRNA and the protein level already after 6 h of treatment and remained high for all the duration of the experiment in both cell lines. Although cultured cells are not an excellent model to appreciate variations in adhesion molecules, following treatment with kynurenine, in FTC-133 cells, we observed a decrease in E-cadherine (epithelial marker) mRNA levels and a specular increase in mRNA levels of two markers of mesenchymal cells, namely N-cadherine and fibronectin-1. Similarly, BcPap cells showed increase in the mesenchymal markers (N-cadherine and fibronectin-1) but no detectable expression of E-cadherine, neither in basal condition or after kynurenine administration.

Altogether, the obtained data suggested that kynurenine-driven activation of AhR might play an oncogenic function not only through the induction of immune tolerance, but also through the initiation of EMT.

Kynurenine treatment induced an increase in OCT4 expression only in FTC-133 cells whereas in BcPap cells a significant difference with untreated cells could not be found. It is known that when AhR is activated by high affinity ligands, such as 2-(1′*H*-indole-3′-carbonyl)-thiazole-4-carboxylic acid methyl ester (ITE), binds to OCT4 promoter and inhibits the transcription of the stemness marker. However, the consumption of tryptophan and the accumulation of the low affinity ligand kynurenine in the tumor microenvironment, together with hypoxia, may cause an increase in OCT4 levels [21]. It is possible that in FTC-133 cells, in which the presence of active IDO1 cause the consumption of tryptophan and the accumulation of kynurenine, AhR is not able to bind efficiently the promoter of the OCT4 gene, producing the observed increase when exogenous kynurenine is added to the medium. Further studies will be needed to clarify this interesting aspect.

Finally, using a wound-healing assay, we demonstrated that kynurenine-mediated AhR activation significantly increased the ability of FTC-133 cells to migrate and close the wound. The same effect could be detected in BcPap cells, but without reaching statistical significance. Moreover, in both cell lines, CH223191 reduced significantly the percentage of wound closure compared with parental cells, probably blocking endogenous AhR activity. The addition of kynurenine to CH223191 did not affect significantly the percentage of closure compared to cells treated with CH223191 alone. Kynurenine-induced AhR activation increased the invasiveness in BcPap cells that was abolished by CH223191 administration. In FTC-133 cell line this capability did not appear to be mediated by kynurenine. This different behavior was probably due to a greater expression of MMP1 in BcPap than in FTC-133. Moreover, BcPap cells harbor BRAFV600E mutation that is known to favor MMPs expression [22].

Overall, these data suggested an involvement of kynurenine-induced AhR activation in conferring a more aggressive phenotype to thyroid cancer cells, by contributing to the onset of an immune-tolerant microenvironment and promoting cellular migration and invasiveness.

## 4. Materials and Methods

### 4.1. Tissue Samples and Patients

We examined surgical specimens of 90 PTCs, 11 MTCs, and six ATCs. All tissues were snap frozen at the time of surgery and stored at −80 °C until use. The presence of autoimmune chronic thyroiditis was assessed, looking for a positive antithyroperoxidase antibody title in patients’ medical charts and/or reviewing histological sections of surgical specimens. The study was approved by the local medical ethics committee (N. 23665/10/AV of 01/26/10). Each study participant provided written informed consent to the collection of fresh thyroid tissue for genetic studies. All the tumors were genetically characterized (PTCs: BRAF, RAS and RET/PTCs; ATCs: BRAF and RAS; MTCs: RET and RAS) and the results were previously published [14].

### 4.2. Transgenic Mice Tissue Samples

AhR expression was evaluated by IHC in thyroid cancer samples derived from transgenic mice, characterized by conditional expression of BRAFV600E in the thyroid. We analyzed slides from 14 thyroid cancers, 4 normal thyroids and 2 lymph nodal metastases from 3 different mouse-models created in Dr. James Fagin’s laboratories (Memorial Sloan Kettering Cancer Center, New York) and kindly provided by Dr. Jeffrey Knauf (Memorial Sloan Kettering Cancer Center, New York) [17].

In detail, AhR expression was analyzed in 6 thyroid tumors and 2 lymph node metastases from mice characterized by doxycycline (dox)-inducible BRAFV600E expression in the thyroid in a p53-/- background (TetOn-BRAF-P53). These mice developed high penetrant and poorly differentiated cancers [23]. To induce BRAFV600E expression, 4 weeks old mice were treated with dox for 6 to 10 weeks and treatment was stopped at 0 to 7 weeks before sacrifice, as indicated in Table 1 (Table 1).

We analyzed AhR expression in 6 thyroid tumors that carried BRAFV600E mutation in heterozygosis, and 3 normal thyroid samples (BRAFWT), derived from a BRAF thyroid-specific knock-in model (BRAF-Lox/TPO-Cre) [24]. These mice developed papillary-like tumors, when BRAFV600E was present in heterozygosis. Mice were sacrificed at 10 to 36 weeks of age, as indicated in Table 2.

Finally, we analyzed 2 thyroids of mice treated for 7 days with dox and one not treated with dox (control) coming from mice characterized by dox-inducible BRAFV600E expression in the thyroid in a p53 WT background (Tg-rtTA/tetO-BRAFV600E). This model made it possible to study the first stages of BRAFV600E-mediated transformation [25].

### 4.3. Cell Cultures

The BcPap cell line (derived from a PTC) was acquired from DSMZ (DSMZ-Deutsche Sammlung von Mikroorganismen und Zellkulturen—German Collection of Microorganisms and Cell Cultures) and was grown in Roswell Park Memorial Institute (RPMI) 1640 medium supplemented with 10% fetal bovine serum (FBS; Life Technologies) [26]. The TPC-1 cell line (derived from a PTC) was provided by Professor Alfredo Fusco (University of Naples, Naples, Italy) and was grown in Dulbecco’s Modified Eagle Medium (DMEM) medium supplemented with 10% FBS [27]; authentication included detection of RET/PTC1 [28]. The C643 cell line (derived from an ATC) was provided by Professor Niels Heldin (University of Uppsala, Uppsala, Sweden) and was grown in RPMI 1640 medium supplemented with 10% FBS [29]. The 8505c cell line (derived from an ATC) was provided by Dr Carmelo Nucera (Harvard Medical School, Boston, MA, USA) and was grown in RPMI 1640 medium supplemented with 10% FBS [30]; authentication was conducted by DNA profiling at University of Colorado Cancer Center DNA Sequencing and Analysis Core (Aurora, Colorado) [28]. The FTC-133 cell line (derived from a poorly differentiated human thyroid carcinoma) was provided by Professor Diego Russo (University of Catanzaro, Catanzaro, Italy) and was grown in DMEM/F12 medium supplemented with 10% FBS [31]; authentication was conducted by DNA profiling at University of Colorado Cancer Center DNA Sequencing and Analysis Core (Aurora, Colorado) [28]. The Cal62 cell line (derived from an ATC) was acquired from DSMZ and was grown in DMEM medium supplemented with 10% FBS [32]. To evaluate the effect of kynurenine on gene expressions, FTC-133 and BcPap cells were seeded in 100 mm plates and treated with kynurenine (100 µM in PBS or in DMSO as indicated) and/or CH223191 (10 µM in DMSO) when an average confluence of 80% was reached. The cells were cultured for the indicated times (6 h; 18 h or 24 h) before harvesting for RNA or protein extraction. Kynurenine and CH223191 were purchased from Sigma-Aldrich (St. Louis, MO, USA).

### 4.4. Quantitative PCR

Total RNA was extracted from tissues and cells with Trizol (Life Technologies), and first-strand cDNA synthesis was performed using 2 µg of each RNA sample primed with random hexamers with 200 U of Superscript III reverse transcriptase (Life Technologies), according to the manufacturer’s instructions. To evaluate expression levels of AhR, CYP1A1 and IDO1 in tumor and/or cell samples, a quantitative PCR Assays-on-Demand gene kit was used (assay identification AhR: Hs00169233_m1; CYP1A1: Hs 00153120_m1; IDO1: Hs00158032_m1, Applied Biosystems), and *β*-actin was used as endogenous control (predeveloped Taq-Man assay reagents VIC dye-labeled; Applied Biosystems), as described previously [33]. In detail, all amplification reactions were performed in triplicate and the threshold cycles averaged. Results (determined with the 2^−ΔΔCT^ method) were normalized to a commercial cDNA sample of human normal thyroid (BD Biosciences CLONTECH), derived from 65 normal tissues, that was used as normal reference. The data are presented as RQ obtained normalizing the acquired data with those of the normal thyroid. To evaluate expression levels of CYP1B1, SLUG, E-Cadherin, N-Cadherin, fibronectin-1, OCT4, MMP1, MMP2 and MMP9 quantitative PCR amplifications were performed using Platinum SYBR greenER quantitative PCR Super Mix UDG Universal (Life Technologies) according to the manufacturer’s instructions. The sequences of the PCR primers are shown in Table 3, and the results were analyzed as described previously [34]. In detail, the cycle threshold value, coupled with individualized amplification efficiencies for each primer set, was used to calculate the normalized expression of the indicated gene mRNA using the Q-Gene software [35]. A commercial cDNA sample of human normal thyroid (BD Biosciences CLONTECH), derived from 65 normal tissues, was used as normal reference and the raw mRNA expression data are presented as arbitrary units.

### 4.5. Immunohistochemistry

Immunohistochemistry was performed on human PTC tissue sections and mice tissues using the primary monoclonal anti-human-rat-mouse AhR antibody diluted 1:250 (clone RPT1, Thermo Fisher Scientific, Waltham, MA USA), as described previously [36]. In detail, AhR staining intensity of tumor samples was compared to that of corresponding adjacent normal tissues. To judge the results, semiquantitative methods were adopted. In detail, AhR IHC score was calculated by combining the staining intensity with the percentage of immunoreactive cells. Staining intensity was rated on a scale of 0–3 (0, negative; 1, weak; 2, moderate; and 3, strong). Each tumor was then scored for the percentage of immunoreactive cells. The immunohistochemical score was then assigned to each tumor by multiplying the percentage of positive cells for the staining intensity. The IHC score ranged from 0 to 300.

The cell blocks were prepared employing the CellientTM Automated System. The formalin-fixed samples were previously centrifuged at 1727 rpm for 10 min and the supernatant was removed. The remnant material was placed in modified biological cassettes and underwent a vacuum-assisted filtration. Afterwards, the cells were deposited in a uniform layer until the filter is saturated. Eosin stain was added, and the materials firstly went through absolute alcohol and secondly through xylene; finally, it was molten in paraffin. Once this last hardens, the filter pulls away and the cells were in one plane in the wax. As a result, sections of 4 µm were performed and placed on slides with permanent positive charged surface. The immunostaining for AHR were performed by BOND-III fully automated IHC stainer (Leica Biosystems, Wetzlar, DE), using the anti-AHR antibody (clone RPT1, dilution 1:250, ThermoFisher Scientific, Waltham, MA USA). AhR staining in cells was evaluated assigning staining intensity (0, negative; 1, weak; 2, moderate; and 3, strong).

### 4.6. Immunoblotting Experiments

Immunoblotting experiments were performed according to standard procedures. Polyclonal anti-human AhR antibody was purchased from Cell Signaling and used at a 1:1000 dilution, monoclonal anti-human IDO1 antibody (clone 1F8.2) was purchased from Millipore and used at a 1:1000 dilution, monoclonal anti-human SLUG antibody (clone C19G7 Cell Signaling) and monoclonal anti-human tubulin (clone DM1A) was purchased from Sigma and used at a 1:5000 dilution. Secondary anti-mouse antibody coupled to horseradish peroxidase was purchased from Sigma.

### 4.7. Evaluation of Cellular Migration and Invasiveness

Cell migration was evaluated using The Oris™ Universal Cell Migration Assembly Kit (Platypus Technologies, Madison, WI, USA), according to the manufacturer’s instructions. In details, 1 × 10^4^ FTC-133 or 3 × 10^4^ BcPap cells were seeded in 96-wells plate assembly with Oris™ Cell Seeding Stoppers in 100 µL of complete medium and plates were incubated 24 h to permit cell attachment before removing the stoppers. At confluence, medium was removed to eliminate any unattached cells and replaced with fresh medium with kynurenine (100 µM) and/or CH223191 (10 µM). Treatments were renewed after 8 h, and the images were acquired after 16 h–24 h using a microscope (Olympus IX51) assembly with camera (Olympus, Hamburg, DE). Samples were repeated six times in each experiment and three independent experiments were performed. The unclosed area was compared with that of reference wells, in which stoppers were removed only at the time of acquisition. Images were analyzed using Image J and the percentage of closure in treated cells was compared with those of parental cells.

Cell invasiveness was evaluated using a Beyond chamber assay with 8 µM polycarbonate membrane transwell permeable supports in 24-well plates (Costar, Corning, NY, USA) pretreated with 100 µL ECM Gel Matrix (Sigma Aldrich, St. Louis, MO, USA) for 30 min at 37 °C. One hundred thousand FTC-133 or BcPap cells were seeded at the top of the membrane in a serum-free medium and treated with kynurenine (100 µM) and/or CH223191 (10 µM). Treatments were renewed every 8 h. The bottom wells were filled with 600 µL of complete medium (10% FBS). After 48 h ECM matrix was removed and invasive cells, both inside the polycarbonate membrane and in the lower chamber, were fixed with 70% ethanol, stained with 0.2% crystal violet in a 12 mM methanol solution and acquired with an optical microscope (Olympus IX51) assembly with camera (Olympus). We performed three independent experiments each in duplicates.

### 4.8. Statistical Analysis

Statistical analysis was performed using Predictive Analytic Software release 17.0.2 (SPSS Inc., IBM Chicago, IL, USA). The adopted techniques included the unpaired Student’s *t* test, the one-sample *t* test, the Mann-Whitney U nonparametric test, and Spearman’s rho, as appropriate and as indicated. All differences were considered significant when *p* < 0.05.

## 5. Conclusions

In conclusion, these data suggested a pivotal role of AhR in thyroid cancer tumorigenesis and confirmed the importance of the IDO1-kynurenine-AhR pathway not only in mediating an immunosuppressive microenvironment but also in favoring the acquisition of a mesenchymal phenotype that could promote invasiveness and metastasis. Although further study is needed, these data suggest the potential importance of the inhibition of this pathway for the treatment of advanced, metastatic or radioactive iodine-refractory thyroid carcinomas either as single approach or in combination with other therapies such Tirosine Kinase Inhibitors or immunotherapy.

## Figures and Tables

**Table 1:** Schedule of BRAFV600E dox-induction in TetON-BRAF-P53 mice.

Mice Model: TetOn-BRAF-P53					
ID	Sex	Analyzed Tissue	Dox Treatment (Weeks)	Dox Withdrawal (Weeks)	Age at Sacrifice (Weeks)
**B101**	F	Thyroid Cancer	6	0	10
**B101L**	F	Lymph node metastasis	6	0	10
**A3858**	F	Thyroid Cancer	7	7	20
**A3624**	M	Thyroid Cancer	5	0	11
**A8717**	M	Thyroid Cancer	8	3	17
**A8742L**	M	Lymph node metastasis	7	4	17
**A2492**	M	Thyroid Cancer	6	0	13
**B246**	M	Thyroid Cancer	10	1	16

**Table 2:** Features of analyzed BRAF-Lox/TPO-Cre. HET: heterozygous; WT: wild type.

Mice Model: BRAF-Lox/TPO-Cre				
ID	Sex	BRAF Genotyping	Histotype	Age at Sacrifice (Weeks)
**6135**	M	HET	PTC	10
**2790**	M	WT	NT	33
**2731**	M	HET	PTC	35
**4826**	F	WT	NT	19
**4816**	F	HET	PTC	21
**4820**	F	HET	PTC	20
**2811**	F	WT	NT	31
**2786**	F	HET	PTC	36
**2787**	F	HET	PTC	36

**Table 3:** Primers used for the real-time quantitative PCRs. The sense primer is listed first and the antisense primer second.

	Primer Sequence (5′-3′)
Β-Actin	GCACAGAGCCTCGCCTTTGCC ATGCCCACCATCACGCCCTGG
CYP1B1	GGCTGGATTTGGAGAACGTA CTCGAGTCTGCACATCAGGA
E-Cadherin	CCCAGGAGCCAGACACATTT TTAGGGCTGTGTACGTGCTG
Fibronectin 1	GAGATGAAACCTGAAGCTGA ACCAATCTTGTAGGACTGAC
MMP1	AGTCCAGAAATACCTGGAAAAATAC CCACATCAGGCACTCCACAT
MMP2	GCTACGATGGAGGCGCTAAT TCAGGTATTGCACTGCCAACT
MMP9	CTGGAGGTTCGACGTGAAGG AGCGGTCCTGGCAGAAATAG
N-Cadherin	GGACCGAGAATCACCAAATG CTTGAGGTAACACTTGAGGG
OCT4	GAGTGAGAGGCAACCTGGAG ACACTCGGACCACATCCTTC
SLUG	GACCCTGGTTGCTTCAAGGA GAATGGGTCTGCAGATGAGC
